# Reintervention for Failed Aortic Bioprostheses: Distinct Patient Profiles for Redo Surgery and Valve-in-Valve TAVR in an All-Comers Cohort

**DOI:** 10.3390/jcm15020474

**Published:** 2026-01-07

**Authors:** Daniela Geisler, Zsuzsanna Arnold, Marieluise Harrer, Rudolf Seemann, Georg Delle-Karth, Martin Grabenwöger, Markus Mach

**Affiliations:** 1 Department of Cardiac and Vascular Surgery, Clinic Floridsdorf, 1210 Vienna, Austria; zsuzsanna.arnold@gesundheitsverbund.at (Z.A.); marieluise.harrer@gesundheitsverbund.at (M.H.); martin.grabenwoeger@gesundheitsverbund.at (M.G.); 2 Institute of Head and Neck Diseases, Evangelical Hospital, 1180 Vienna, Austria; rudolf.seemann@gmail.com; 3 Department of Cardiology, Clinic Floridsdorf, 1210 Vienna, Austria; georg.delle-karth@meduniwien.ac.at; 4 Karl Landsteiner Institute of Cardiovascular Research, 1210 Vienna, Austria; 5 University Clinic of Cardiac and Thoracic Aortic Surgery, Medical University Vienna, 1090 Vienna, Austria; markus.mach@meduniwien.ac.at; 6 Department of Cardiac Surgery, University Hospital Graz, 8036 Graz, Austria

**Keywords:** redo SAVR, bioprosthesis, ViV TAVI, transcatheter aortic valve replacement, reintervention, bioprosthetic valve failure

## Abstract

**Background/Objectives**: Aortic valve therapy increasingly follows a lifetime management concept. As all bioprostheses ultimately degenerate, optimal outcomes rely on the appropriate selection and timing of treatment modality. This study evaluates outcomes of redo surgical aortic valve replacement (redo-SAVR) and valve-in-valve transcatheter aortic valve replacement (ViV-TAVR) in a consecutive, unselected real-world cohort treated for bioprosthetic valve failure (BVF). **Methods**: A single-center retrospective analysis of all patients undergoing redo-SAVR or ViV-TAVR for BVF between June 2019 and December 2024 was conducted. The primary endpoint was survival at 30 days and at 1, 3, and 5 years; the secondary endpoint was time to reintervention. Cox proportional hazards models were used; proportionality was tested; subgroups were defined by indication and presence of concomitant procedures. **Results**: Eighty-three patients were included (redo-SAVR *n* = 42; ViV-TAVR *n* = 41). All active endocarditis cases were managed surgically. In isolated procedures, 30-day survival was 95.5% after redo-SAVR (100% when excluding endocarditis) and 100% after ViV-TAVR; 5-year survival was 81.3% and 94.1%, respectively (94.4% for isolated redo-SAVR excluding endocarditis). Because hazards were non-proportional and risk sets were sparse beyond 5 years, we fitted a time-split Cox model (0–5 years). In multivariable analysis, endocarditis (HR 4.45, 95% CI 1.16–17.04) and NYHA IV (HR 4.87, 95% CI 0.98–24.17)—not treatment modality—were associated with mortality. **Conclusions**: In a real-world, all-comers setting, early outcomes for isolated reinterventions were favorable with both pathways. Mortality patterns were case-mix driven—especially by endocarditis and the need for concomitant surgery. Accordingly, ViV-TAVR and redo-SAVR should be viewed not as competing procedures but as complementary, scenario-specific options within a lifetime management strategy.

## 1. Introduction

In recent years, an increasing number of patients—including younger individuals—with aortic stenosis have opted for bioprosthetic heart valves (BHVs), prioritizing quality of life despite the potential need for future reinterventions [1,2,3]. In parallel, transcatheter aortic valve replacement has evolved from a therapy for high-risk candidates to a widely accepted standard of care [4]. These trends bring lifetime management to the forefront: rather than a single procedure, the sequence of interventions across the lifespan must be planned [5,6,7].

Surgical AVR (SAVR) remains the most established index strategy, followed by reoperation or serving as a platform for future valve-in-valve (ViV) procedures. In contrast, initial TAVR—while less invasive—may restrict options if repeat TAVR is not feasible and surgical explantation is required [8,9,10,11].

Regardless of strategy, BHVs are inherently prone to structural degeneration and ultimately fail [6]. ViV-TAVR has gained momentum as a less invasive alternative to redo-SAVR [6,12,13]. Large meta-analyses show early and intermediate advantages for ViV-TAVR, whereas longer-term survival appears to favor redo-SAVR [12,14,15]. However, real-world applicability is limited because many pooled cohorts focused on elective structural valve deterioration (SVD) and excluded prosthetic valve endocarditis (PVE) and concomitant surgery [4,16,17]. To address these gaps, we included a consecutive, all-comers cohort of patients with bioprosthetic valve failure (BVF) undergoing redo-SAVR or ViV-TAVR, with a follow-up of up to 5 years. By including the full spectrum of indications, including endocarditis, and the need for concomitant procedures, our study complements current evidence. It delineates the clinical contexts in which each strategy functions as a complementary component of lifetime valve management rather than a competing alternative.

## 2. Materials and Methods

### 2.1. Study Cohort

All patients who underwent ViV-TAVR or redo-SAVR for bioprosthetic valve dysfunction between June 2019 and December 2024 at the Department of Cardiac and Vascular Surgery and the Department of Cardiology, Clinic Floridsdorf, Vienna, were retrospectively analyzed. Bioprosthetic valve dysfunction was defined according to VARC-3 criteria as SVD, non-structural valve dysfunction, thrombosis, and endocarditis [18]. Multidisciplinary heart-team decisions (including cardiac surgery, interventional cardiology, cardiac imaging specialist) adhered to contemporary ESC/EACTS guidance and considered patient risk/frailty and anatomical suitability. ViV-TAVR was selected for BVF without active infection in patients with elevated surgical risk (per EuroSCORE II) and of older age, while active PVE and cases requiring concomitant procedures were treated with redo-SAVR [19].

The study protocol was approved by the local ethics committee (EK 24-205-VK). Patient consent was waived, due to the retrospective character of the study. Patients were excluded if they had a prior mechanical aortic valve prosthesis or Ross procedure. Retrospective data were retrieved from the institutional database, which is continuously updated as part of the Austrian national quality assessment program. Mortality data were obtained through electronic health records, obituary notices, and telephone follow-up.

### 2.2. Endpoints

The primary endpoint was survival at 30 days, 1, 3, and 5 years. The secondary outcome was time to reintervention, defined as the time from the index surgery to the date of reintervention. Additionally, the underlying mechanisms of BVF were assessed.

### 2.3. Statistical Analysis

All statistical analyses were conducted using RStudio (version 4.5.1). Survival was estimated using the Kaplan–Meier method (numbers at risk shown; log-rank *p* values reported for main comparisons). Cox proportional hazards models were fitted to estimate hazard ratios (HRs) with 95% confidence intervals (CIs). The proportional hazards (PH) assumption was assessed using Schoenfeld residuals. Because ≤2 patients per group remained at risk beyond 5 years, we prespecified time-restricted (0–5-year) Cox models and interpret HRs as average early-period effects, alongside unadjusted KM/log-rank results. Treatment was coded as four strata: isolated redo-SAVR (redo-iso), redo-SAVR with concomitant surgery (redo-com), isolated ViV-TAVR (ViV-iso; reference), and ViV-TAVR with concomitant procedure (ViV-com). Within 0–5 years, the four-level group model did not reject proportionality (global χ^2^ = 5.43, df = 3, *p* = 0.14). Univariable models were fit for group, endocarditis, and NYHA IV, followed by a multivariable model including these covariates; covariate-specific PH tests were non-significant (group *p* = 0.193; endocarditis *p* = 0.167; NYHA IV *p* = 0.287), whereas the global test indicated modest residual non-proportionality (χ^2^ = 14.44, df = 5, *p* = 0.013). Accordingly, HRs are reported as average effects over 0–5 years. The ViV-com stratum had no deaths ≤ 5 years; its coefficient was therefore non-estimable and is reported descriptively. All tests were two-sided (α = 0.05). For baseline characteristics, continuous variables were compared with the Wilcoxon rank-sum test; categorical variables with Pearson’s χ^2^ (Fisher’s exact when expected counts < 5); ordinal variables with a χ^2^ test for trend.

## 3. Results

### 3.1. Primary Endpoint

A total of 83 patients (50 male, 33 female) with a dysfunction of the aortic valve bioprosthesis underwent either redo-SAVR (*n* = 42) using a next-generation aortic bioprosthesis or ViV-TAVR (*n* = 41) with self- or balloon-expandable valves via transfemoral or transapical access (see Supplementary Table S1). All active endocarditis cases were managed with redo-SAVR, often with concomitant procedures; no patient with suspected or confirmed active endocarditis underwent ViV-TAVR in this series. Demographic characteristics of the two groups (redo-SAVR and ViV-TAVR) are presented in Table 1. Surgical-related data are shown in Table 2.

**Figure 1:**
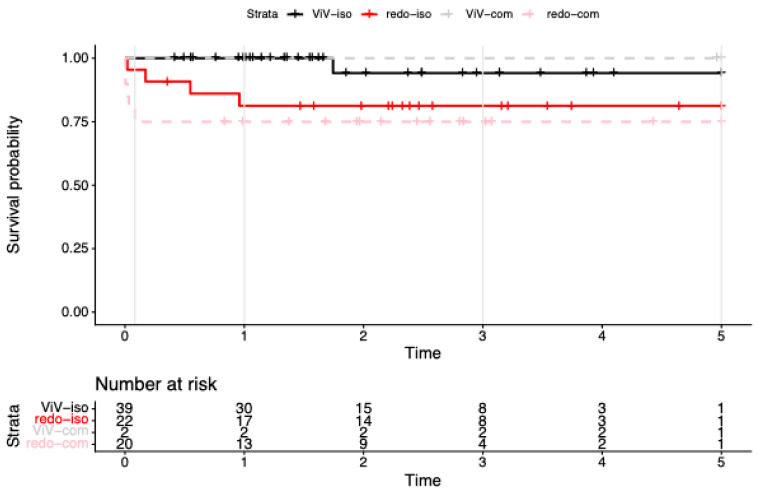
Kaplan–Meier survival to 5 years after reintervention for failed bioprosthetic aortic valves. Kaplan–Meier survival curves for redo surgical aortic valve replacement (redo-SAVR) and valve-in-valve transcatheter aortic valve replacement (ViV-TAVR), shown separately for isolated procedures (iso) and procedures with concomitant surgery (com). Endocarditis cases are included in all strata. Numbers at risk are displayed below the *x*-axis; tick marks indicate censoring. By design (2019–2024 accrual), >5-year data were sparse; we therefore report period-specific (0–5 y) Cox estimates.

Cumulative survival in the isolated redo-SAVR group (*n* = 22) was 95.5% (95% CI: 87.1–100.0%) at 30 days and 81.3% (95% CI: 66.4–99.7%) from 1 to 5 years (Figure 1). Excluding four patients with endocarditis from the isolated redo-SAVR group, the picture changed to a 100.0% (95% CI: 100.0–100.0%) 30-day survival and 94.4% (95% CI: 84.4–100.0%) at 1-to-5-year follow-up. One of the four patients with endocarditis in the isolated redo group died within the first 30 days (cumulative survival rate (CSR) = 75.0%, 95% CI: 42.6–100.0%), and two died within the first year (CSR = 25.0%, 95% CI: 4.6–100.0%).

Among patients undergoing redo-SAVR with concomitant procedures (*n* = 20), 30-day survival was 75.0% (95% CI: 58.2–96.6%), with no additional deaths observed during the subsequent 5-year follow-up. When excluding the seven patients with endocarditis, survival outcomes remained stable at 76.9% (95% CI: 57.1–100.0%) over the entire period.

In the isolated ViV-TAVR group (*n* = 38), 30-day and 1-year survival was 100%, and 94.1% (95% CI: 83.6–100%) at 3–5 years. Notably, two patients in the ViV-TAVR cohort underwent concomitant procedure (mitral valve replacement) via transapical access. Both survived the entire follow-up period (4.89 and 5.04 years, respectively).

Because few patients remained at risk beyond 5 years, Cox models were restricted to 0–5 years (*n* = 83; events = 10). With the treatment factor coded as four strata—isolated ViV-TAVR (ViV-iso, reference), ViV-TAVR with concomitant surgery (ViV-com), isolated redo-SAVR (redo-iso), and redo-SAVR with concomitant surgery (redo-com)—the overall group effect was significant by likelihood-ratio test (χ^2^ = 8.09, df = 3, *p* = 0.04; concordance = 0.76). Relative to ViV-iso, redo-iso showed a higher, borderline hazard (HR 6.98, 95% CI 0.78–62.52; *p* = 0.083), and redo-com had a significantly higher hazard (HR 10.89, 95% CI 1.27–93.32; *p* = 0.029). The ViV-com subgroup had no deaths ≤ 5 years, yielding a non-estimable (infinite) coefficient (“complete separation”); this stratum is therefore reported as not estimable (NE) and interpreted descriptively. Proportional hazards for the 4-level model in this window did not reject (global cox.zph χ^2^ = 5.43, df = 3, *p* = 0.14; see Table 3).

In univariable models, endocarditis (HR 7.89, 95% CI 2.27–27.47; *p* = 0.001) and NYHA IV (HR 5.99, 95% CI 1.26–28.43; *p* = 0.024) were associated with higher mortality.

**Figure 2:**
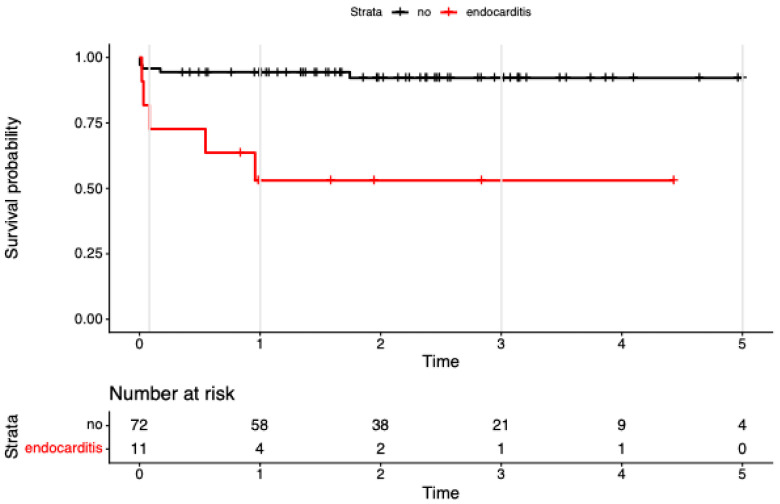
Kaplan–Meier survival to 5 years after reintervention for failed bioprosthetic aortic valves, stratified by infective endocarditis at the time of redo. Kaplan–Meier survival to 5 years after reintervention for failed bioprosthetic aortic valves, stratified by infective endocarditis at the time of redo (endocarditis *n* = 11; no endocarditis *n* = 72). Censoring shown by plus signs; numbers at risk displayed below; follow-up truncated at 5 years. Univariable Cox: endocarditis associated with higher mortality (HR ≈ 7.9; 95% CI 2.3–27.5; *p* ≈ 0.001.

**Figure 3:**
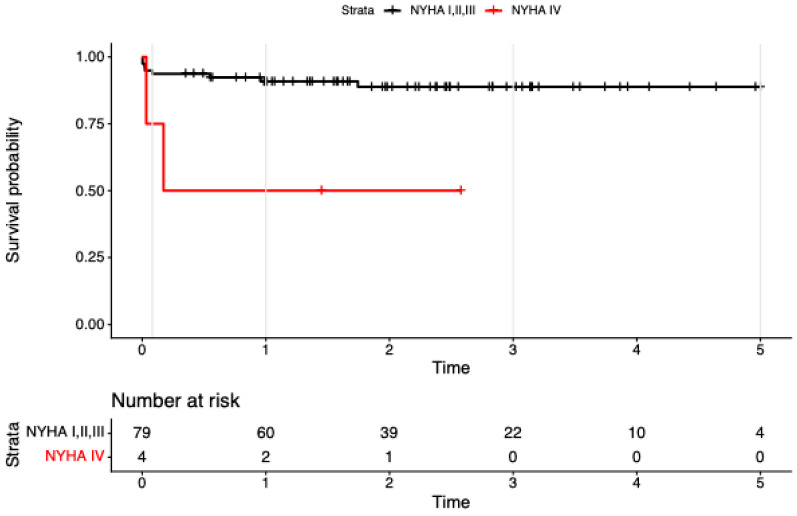
Kaplan–Meier survival to 5 years after reintervention for failed bioprosthetic aortic valves, stratified by baseline NYHA class (I–III vs. IV). Tick marks show censoring; numbers at risk are displayed beneath the *x*-axis (NYHA I–III: 79, 60, 39, 22, 10, 4; NYHA IV: 4, 2, 1, 0, 0, 0). Follow-up is administratively truncated at 5 years. Survival is consistently lower in the NYHA IV subgroup.

In the multivariable model including the 4-level treatment factor plus endocarditis and NYHA IV (concordance = 0.84; LR χ^2^ = 14.83, df = 5, *p* = 0.01), the treatment effects attenuated and were no longer significant (vs ViV-iso: redo-iso HR 4.32, 95% CI 0.45–41.09, *p* = 0.203; redo-com HR 4.65, 95% CI 0.42–52.14, *p* = 0.213; ViV-com NE due to zero events). Endocarditis remained associated with mortality at borderline significance (HR 4.32, 95% CI 0.99–18.86; *p* = 0.052; see Figure 2), and NYHA IV was similarly borderline (HR 4.86, 95% CI 0.98–24.14; *p* = 0.053; see Figure 3). Global PH test showed modest residual non-proportionality; covariate-specific tests were non-significant

### 3.2. Secondary Endpoint

The interval between index surgery and reintervention was significantly shorter in the redo-SAVR group (6.8 ± 4.4 years) compared to the ViV TAVR group (9.8 ± 4.2 years; Wilcoxon rank sum test with continuity correction: W = 559, *p* = 0.006). After exclusion of endocarditis, the interval was 7.8 ± 4.3 years in the redo-SAVR (Wilcoxon rank sum test with continuity correction: W = 498, *p*-value = 0.119.

## 4. Discussion

Managing the aortic valve over a lifetime has moved to the forefront as the use of bioprosthetic valves increases [19]. In this consecutive, all-comers cohort—including infective endocarditis and concomitant surgery—early outcomes for isolated reinterventions were favorable with both strategies. Because enrolment spanned 2019–2024, few patients had >5 years of follow-up; we therefore centered inference on 0–5 years, where risk sets were adequate. In this window, the four-stratum group term (ViV-iso [reference], ViV-com, redo-iso, redo-com) was significant: excess hazard was concentrated in redo-SAVR with concomitant surgery, with only a borderline increase for redo-SAVR isolated versus ViV-iso. The ViV-com subgroup had no deaths ≤ 5 years, yielding a non-estimable coefficient (complete separation). In multivariable analyses adjusting for endocarditis and NYHA IV, treatment effects attenuated and lost statistical significance, whereas endocarditis (and to a lesser extent NYHA IV) remained the principal drivers of mortality. Overall, the mortality patterns appear case-mix driven—particularly by infectious endocarditis and the need for combined surgery—rather than determined by modality per se.

Zero events in ViV-com should not be over-interpreted as protection; they reflect small numbers and selection (very few patients underwent concomitant transcatheter mitral replacement in the ViV pathway). Likewise, wide CIs around the redo estimates reflect low event counts. Within these limits, the data support complementary, scenario-specific use of both strategies: ViV-TAVR performs well for isolated BVF in older or higher-risk patients, whereas redo-SAVR is indispensable when infectious endocarditis control, concomitant repair, or annular enlargement is required to minimize PPM and preserve future ViV options.

Because hazards were non-proportional over the complete follow-up and a few patients remained at risk beyond 5 years, we report period-specific (0–5-year) HRs as average early-period effects and complement inference with KM/log-rank comparisons. This prioritizes validity over extrapolation from unstable late data.

Thirty-day mortality after isolated redo-SAVR (including endocarditis) was 4.5%, consistent with 4.6% in 3380 redo-SAVR cases from the Society of Thoracic Surgeons Adult Cardiac Surgery Database (Kaneko et al.) and with Peterson et al. (5.7%); excluding endocarditis, 30-day mortality was 0%, which we also observed for ViV-TAVR [20,21,22]. At five-years, survival was 94.4% after isolated redo-SAVR (excluding endocarditis) and 94.1% after ViV-TAVR, which compares favorably with the large meta-analysis by Sa et al. 2024, where ViV-TAVI was associated with higher mortality than redo-SAVR (HR 1.92, 95% CI 1.58 to 2.33, *p* < 0.001) and with the PARTNER 2 ViV registry (49.4% mortality at 5 years) [14,23]. Importantly, most large meta-analyses predominantly pooled elective SVD and did not mention endocarditis or combined procedures.

Baseline risk differed significantly between groups. ViV-TAVR recipients were older (79.0 vs. 70.6 years) and more often deemed higher surgical risk by the heart team, consistent with its less-invasive profile and historical use in elderly candidates [1,12,14]. In the redo-SAVR cohort, 47.6% required concomitant procedures and ~26% presented with endocarditis, contributing to a higher EuroSCORE II that narrowly missed significance (*p* = 0.056) [14]. In our series active PVE was managed surgically to enable radical debridement and valve explantation; this aligns with guidance that treats active endocarditis as a relative contraindication to TAVR [19]. While a recent Cleveland Clinic review summarized small series of off-label TAVR in prohibitive-risk patients, evidence remains limited and concerns about persistent infection/relapse persist; such use should be exceptional [24]. Because many prior series excluded endocarditis, real-world complexity is often underrepresented. These case-mix differences reinforce a complementary, not competing, view of modalities [21]. Borderline/challenging cases were decided case-by-case by a multidisciplinary heart team with a lifetime-sequencing lens, integrating indication, anatomy/access, frailty, EuroSCORE II, end-organ function, and preservation of future options. We favored redo-SAVR when infection, concomitant repair, coronary-obstruction risk, or the need for annular enlargement was present.

With respect to durability, prior studies generally report comparable BVF rates for surgical and transcatheter valves, with average durability around 10 years [25]. The shorter interval from index operation to reintervention among patients proceeding to redo-SAVR largely reflects the inclusion of patients with endocarditis and should not be interpreted as inferior durability of the prior surgical valve; it reflects the timing of the indication [26,27]. Despite growing interest in lifetime management, most available data still emphasize short-term outcomes; robust mid- to long-term evidence remains limited [12,14,21,28].

Anatomy is pivotal when planning reintervention [29]. Key considerations include coronary ostial height, suitability for transfemoral access, annular dimensions, and sinuses of Valsalva size [6,30]. Valve size is particularly determinative of late outcomes: Bleiziffer et al. reported worse ViV-TAVR results with small index valves [31]. In our cohort, over one-third had an index prosthesis ≤ 21 mm; at reintervention, valves ≤ 21 mm were implanted more often with redo-SAVR than with ViV-TAVR, potentially constraining future options and hemodynamics. ViV-TAVR—especially in small annuli—was associated with higher post-procedural gradients, which may increase PPM risk; the long-term clinical impact remains debated [15,28,32,33]. For younger patients, this favors surgical annular enlargement when feasible to upsize the prosthesis, reduce PPM, and preserve the option for later ViV-TAVR [28].

Coronary artery disease (CAD) adds further complexity. CAD and prior CABG have been linked to increased mortality in this setting [14]. As ViV-TAVR adoption widens, maintaining coronary access and the feasibility of repeat percutaneous interventions requires particular attention [5]. In our cohort, baseline CAD and prior CABG were similar across groups; however, a higher proportion of redo-SAVR patients underwent revascularization at the time of reintervention, suggesting a greater burden of complex coronary disease.

Procedure-specific complications also inform selection. Acute kidney injury occurred more frequently after redo-SAVR—consistent with prior reports—underscoring that risk profiles differ and must be integrated into individualized decision-making [16,33].

Taken together, long-term outcomes after BVF reintervention are shaped by interacting factors: procedural choice, anatomic constraints (including valve size), comorbid conditions such as endocarditis and CAD, and patient-specific trajectories. As lifetime management becomes standard, decisions should be individualized from the index procedure onward, with systematic long-term follow-up.

*Limitations.* This single-center retrospective study is modest in size with few events in several strata, limiting precision and precluding propensity matching or formal non-inferiority/equivalence testing. Confounding by indication remains likely because heart-team assignment produced material baseline differences (age, EuroSCORE II, endocarditis, need for concomitant surgery). Time-split Cox modeling (0–5 years) and descriptive handling of the ViV-com stratum (no deaths ≤5 years) were adopted to avoid unstable late estimates. Findings should be interpreted as hypothesis-generating and illustrative of a complementary, not competing, role for the two modalities [34].

*Outlook.* Confirmation will require larger, prospective, multicentre studies with standardized adjudication of endocarditis and concomitant procedures, detailed CT/anatomical and coronary-access assessment, and longitudinal haemodynamics.

## 5. Conclusions

Assessment of long-term outcomes after second valve implantation is essential—particularly in younger patients, for whom durability and the likelihood of future reintervention are central. Our findings support tailored reintervention strategies guided by anatomy, procedural risk, comorbidity profile, and anticipated lifetime needs. Importantly, ViV-TAVR and redo-SAVR are complementary, not competing, modalities within a lifetime management strategy, tailored to the individual patient and clinical context.

## Figures and Tables

**Table 1:** Patient Baseline Characteristics. W—Wilcoxon rank sum test with continuity correction; C—Pearson’s Chi-squared test.

	Redo SAVR (*n* = 42)	ViV TAVR (*n* = 41)	*p*-Test
Age (mean (SD))	70.6 (8.75)	79.0 (5.3)	<0.001 W
Sex, female (%)	18 (42.9)	15 (36.6)	0.719 C
EuroSCORE II (mean (SD))	14.8 (16.2)	8.9 (11.1)	0.056 W
Weight, kg (mean (SD))	75.2 (16.2)	85.1 (21.4)	0.020 W
Height, cm (mean (SD))	169.4 (9.26)	168.8 (16.8)	0.860 W
BMI, (mean (SD))	26.2 (5.1)	34.3 (39.1)	0.185 W
CABG history (%)	7 (16.7)	9 (22.0)	0.740 C
Pacemaker (%)	7 (16.7)	6 (14.6)	1.000 C
Smoking history (%)	6 (14.3)	13 (31.7)	0.104 C
Diabetes (%)	13 (31.0)	17 (41.5)	0.442 C
Dyslipidemia (%)	26 (61.9)	38 (92.7)	0.002 C
Creatine level preoperatively, mg/dL (mean (SD))	1.16 (0.57)	1.06 (0.37)	0.315 W
Dialysis preoperatively (%)	1 (2.4)	0 (0.0)	1.000 C
Hypertension (%)	28 (66.7)	36 (87.8)	0.042 C
Endocarditis (%) Active Treated	11 (26.2) 10 (90.9) 1 (9.4)	0 (0.0) 0 (0.0) 0 (0.0)	0.001 C
Chronic lung disease (%) No Gold 1 Gold 2 Gold 3	37 (88.1) 1 (2.4) 2 (4.8) 2 (4.8)	35 (85.4) 2 (4.9) 4 (9.8) 0 (0.0)	0.385 C
Peripheral vascular disease (%)	2 (4.8)	4 (9.8)	0.649 C
Cerebrovascular disease (%)	3 (7.1)	8 (19.5)	0.181 C
Coronary artery disease (%)	15 (35.7)	17 (41.5)	0.755 C
Left ventricular ejection fraction, % (mean (SD))	56.3 (8.54)	54.08 (9.71)	0.281 W
Reason for reintervention (%) Aortic regurgitation Infectious endocarditis Paravalvular leak Structural valve deterioration	1 (2.4) 10 (24.4) 4 (9.8) 26 (63.4)	0 0 0 41 (100)	0.004 C

BMI—body mass index, CABG—coronary artery bypass grafting, SD—standard deviation.

**Table 2:** Intraoperative Characteristics and Postoperative Outcomes. W—Wilcoxon rank sum test with continuity correction; C—Pearson’s Chi-squared test.

	Redo SAVR (*n* = 42)	ViV TAVR (*n* = 41)	*p*-Test
Hospital stay, days (mean (SD))	21.1 (11.8)	16.1 (14.3)	0.005 W
Aortic valve size, mm (%) 19 20 21 23 25 26 27 29	5 (11.9) 0 (0.0) 17 (40.5) 11 (26.2) 5 (11.9) 0 (0.0) 4 (9.5) 0 (0.0)	0 (0.0) 1 (2.4) 1 (2.4) 18 (43.9) 1 (2.4) 17 (41.5) 1 (2.4) 2 (4.9)	<0.001 C
Index valve size, mm (%) 19 21 23 25 27 29	6 (14.3) 14 (33.3) 10 (23.8) 5 (11.9) 6 (14.3) 1 (2.4)	2 (4.9) 10 (24.4) 17 (41.5) 7 (17.1) 4 (9.8) 1 (2.4)	0.300 C
Procedural duration, min (mean (SD))	353.3 (116.7)	60.8 (15.9)	<0.001 W
IABP (%)	2 (4.8)	0 (0.0)	0.485 C
ECMO (%)	4 (9.5)	0 (0.0)	0.130 C
Combined procedures (%)	20 (47.6)	2 (4.9)	<0.001 C
Concomitant CABG or PCI (%)	5 (11.9)	0 (0.0)	0.069 C
Aortic annular enlargement (%)	7 (16.6)	0 (0.0)	0.019 C
Mitral valve procedure (%)	5 (11.9)	2 (4.9)	0.449 C
Tricuspid reconstruction (%)	2 (4.9)	0 (0.0)	0.485 C
Ascending aortic procedure (%)	6 (14.3)	0 (0.0)	0.037 C
ICU stay, days (mean (SD))	7.8 (10.5)	1.3 (4.8)	<0.001 W
Reoperation for bleeding/tamponade (%)	4 (9.5)	1 (2.4)	0.371 C
Transient neurologic deficit (%)	0 (0.0)	1 (2.4)	0.990 C
Acute kidney injury (%)	9 (21.4)	1 (2.4)	0.020 C
Dialysis/hemofiltration (newly required) (%)	6 (14.3)	2 (4.9)	0.280 C
Permanent pacemaker implantation (%)	6 (14.3)	3 (7.3)	0.504 C
Pneumonia (%)	4 (9.5)	1 (2.4)	0.830 C
Acute limb ischemia (%)	1 (2.4)	1 (2.4)	1.000 C
Mean pressure gradient postoperative (mean (SD))	11.7 (3.1)	16.6 (7.2)	0.015 W
Vmax postoperative (mean (SD))	2.2 (0.3)	2.74(0.6)	0.001 W

IABP—intra-aortic balloon pump, ECMO—extracorporeal membrane oxygenation, CABG—coronary artery bypass grafting, PCI—percutaneous coronary intervention, ICU—intensive care unit, SD—standard deviation.

**Table 3:** Cox proportional hazard models.

Model	Factor	HR	Lower	Upper	*p*-Value
1	combined vs. iso-VIV iso-redo vs. iso-VIV combined redo vs. iso-VIV	0.0 7.0 10.9	0.0 0.8 1.3	Infinity 62.5 93.3	0.083 0.998 0.029
2	Endocarditis	7.9	2.3	27.5	0.001
3	NYHA IV	6.0	1.3	28.4	0.024
4	combined vs. iso-VIV iso-redo vs. iso-VIV combined redo vs. iso-VIV	0.0 4.3 4.7	0.0 0.5 0.4	Infinity 41.1 52.1	0.999 0.203 0.213
	Endocarditis	4.3	1.0	18.9	0.052
	NYHA IV	4.9	1.0	24.1	0.053

## Data Availability

The raw data supporting the conclusions of this article will be made available by the authors on request.

## Supplementary Materials

The following supporting information can be downloaded at https://www.mdpi.com/article/10.3390/jcm15020474/s1, Table S1: Aortic valve implants.

## Abbreviations

The following abbreviations are used in this manuscript:

BVF	bioprosthetic valve failure
BHV	bioprosthetic heart valves
CABG	coronary artery bypass grafting
CAD	coronary artery disease
HR	hazard ratio
PH	proportional hazards
SAVR	surgical aortic valve replacement
TAVR	transcatheter aortic valve replacement
ViV	valve-in-valve
