# Supplementary material for: Reintervention for Failed Aortic Bioprostheses: Distinct Patient Profiles for Redo Surgery and Valve-in-Valve TAVR in an All-Comers Cohort
Source: J Clin Med. 2026 Jan 7;15(2):474. doi: 10.3390/jcm15020474 (PMC12842112; doi:10.3390/jcm15020474)
Supplement: Supplementary file 1 [file jcm-15-00474-s001.zip › jcm-4081285-supplementary.pdf]

Supplementary table S1. Aortic valve implants

Group	Implant	n=	N=
Redo-SAVR	EDWARDS Inspiris Resilia	39	42
	EDWARDS Magna Ease	1	
	EDWARDS Intuity	1	
	CORCYM Perceval	1	
ViV	EDWARDS Sapien 3	12	41
	MEDTRONIC CoreValve Evolut Pro	12	
	MEDTRONIC CoreValve Evolut FX	6	
	EDWARDS Sapien 3 Ultra	3	
	SYMETIS Acurate neo	3	
	ALLEGRA	2	
	SYMETIS Acurate neo2	2	
	ABBOTT Portico	1	
